# Cocaine in Tooth Extraction

**Published:** 1886-05

**Authors:** 


ARTICLE V.

### COCAINE IN TOOTH EXTRACTION.

BY THE EDITOR OF *London Dental Record.*

The remarkable properties of the alkaloid, Cocaine, have, practically, been brought prominently before the medical world only within the past eighteen months. The readers of the *Dental Record* have been kept *au courant* of the chief features of the literature on the subject. During the last year it was very exceptional not to find in every medical periodical some mention made of the drug, and the pages of this journal have contained numerous articles and selections. In that manner has there been presented a sketch of the botany of the cuca plant; a history of the medicinal properties of its leaves; an account of the introduction of the alkaloid, cocaine, as a local anæsthetic; of the physiological action of the drug, as far as has been made out, together with certain preparations and formulæ. In the July number (vol. v., p. 323), mention was first made of the hpyodermic injection of a solution of cocaine for the painless extraction of teeth, painting the gum with it having been found to give but little relief.

Since then Mr. W. A. Hunt, L. R. C. P., &c. communicated to the January number of the *Journal of the British Dental Association* the successful results of his experience. He describes his *modus operandi* as follows: My hypodermic syringe has a capacity of nine minims, and is furnished with a steel needle; it is easily kept sharp with an oilstone, and is better than gold, which too easily becomes blunt. The syringe being filled with hot water, its contents are squirted into a small, short test-tube, at the bottom of which one grain of hydrochlorate of cocaine has been placed. If the salt shows no disposition to dissolve, you can heat the test-tube gently over the spirit lamp until the solution is perfectly clear; then dip in your syringe and take up four minims. Puncture the gum first on the buccal aspect about the centre of the tooth you propose to extract, pressing the needle as vertically as you can, so that its point may reach nearly as far as the apex of the root. The pain of the puncture is usually very slight, and is hardly regarded as the needle passes onward. If the sloping surface of the point is turned towards the alveolus, there is less chance of the bone arresting the onward progress of the needle, and this is the chief difficulty in injecting. Having thrust the needle as far as needed, press the piston; often it will not yield even with force, but if you wait patiently, keeping up firm pressure and perhaps rotating the needle, or even withdrawing it a little, the solution will assuredly flow into the tissues. Keep the needle there half a minute, to prevent the possibility of any of the solution escaping by the puncture. Then re-charge your syringe with the four or five minims of the solution still remaining in the test-tube, and in a similar manner inject deeply the tissues on the lingual side of the tooth. It is remarkable how, when your patient is at the moment suffering from toothache, entire freedom from pain occurs in five or ten seconds after even the first injection.

You have now quickly and deeply injected a strong, hot solution of the agent; the conditions for rapid absorption are thus excellent, and in two minutes, or even less, you can operate with forceps, elevator or splitting forceps, as may be required.

I have never injected less than a grain, but where the solution has flowed out through the puncture, of course there has been a waste of power.

As solutions of this agent do not keep, I have never used anything but a soiution I have prepared myself immediately before the operation, as above described. If you cannot depend upon the accuracy of your chemist, use delicate scales and weigh the cocaine yourself. The grains may be folded in small papers separately, and put in a small stoppered bottle, so that no time may be wasted. I mention accuracy, as, if you take the trouble to weigh reputed grains, you will be astonished what different quantities they sometimes represent.

That the question of using a freshly prepared solution is not a fanciful one, is shown by the discussion at the late meeting of the Ophthalmological Society, where evidence was brought forward by more than one member to prove the occurence of irritation and inflammation after using solutions which had been kept for some time.

Now, with hypodermic injections, this is a danger that must never be lost sight of, and there is good reason for my bringing it forward. Likewise the syringe must be kept scrupulously clean, for it has often to be passed through tissues filled with the products of inflammation; so after use it should be very carefully wiped clean, and I then draw a few drops of liquid carbolic acid up and down the needle, and then wipe it dry.

My opinion is, that where there is much infiltration (by the products of inflammation) in the tissues, a slightly larger dose than a grain may be given. and a minute or so more granted for time for absorption. . . . . I have not observed as yet any constitutional symptoms follow this method, nor have I as yet found the injection to cause any local irritation.

Dr. D. W. Barker, writing in the *Independent Practitioner*, "On The Use of Cocaine by Hypodermic Injection for Extraction of Teeth," says—Complete insensibility to the pain of extraction may be produced by injecting, with an ordinary hypodermic syringe, five drops of a four per cent. solution under the gum, directly over the root of the tooth to be extracted. It will take from five to eight or nine minutes to get the full anæsthetic effect of the Cocaine. Pricking the gum to test its insensibility will indicate when to extract. The extent of insensibility is limited to a small space around the place of injection, and the effect lasts from ten to fifteen minutes, and then pases away.

An important fact to be kept in mind is that the agent is injected directly into the circulation of the patient; hence the drug and its solution should be the purest possible, and the instrument absolutely clean. In order that the solution may be of the best, it should be made of distilled water; all ordinary water contains some organic matter, and a solution made of it will sour in about a week and become not only unfit for use but positively dangerous.

In making the injection care must be taken to inject no air into the tissues; this may be avoided by drawing some of the solution into the syringe, then turning the point upward and expelling the liquid; any air in the syringe will go out first; then fill the syringe as full as required. To avoid running the point against the edge of the alveolus, and also to avoid the thick and somewhat tough margin of the gum, let the point enter the gum an eight of an inch from the margin, and, following the surface of the bone, pass in at least three-eights of an inch; if the bevelled side of the point is held next to the bone it will avoid sticking against it. Press on the piston rod gently and slowly, so as to expell the liquid a drop at a time; inject half of the five drops on one side (buccal or labial) of the tooth, and remaining half on the other (palatine or lingual) side,; hold the syringe point still for half a minute, and then withdraw it slowly, so that the liquid may be taken up by the tissues, and not spurt back when the point is withdrawn. For molars, injection may best be made horizontally, instead of vertically. If there are two or three roots standing close together the injection should be midway between them.

I have been asked if the injection itself does not cause pain. If the gum is first bathed with the solution and the syringe needle is kept very sharp, there need be little cause for complaint. The wound always heals kindly and quickly and there is never any swelling or pain, or any sign of local inflammation or systemic disturbance; the place where the injection is made causes no trouble, but heals with the rest of the wound, and all sign of it disappears.

My own experience fully bears out that of Mr. Hunt, and of Dr Barker. There is always a certain amount of the solution lost into the mouth in the act of injecting, so that of ten minims, having a grain of the alkaloid in solution, probably not more than two-thirds are injected into the tissues of the gum. In one case I took a grain and a half of cocaine, and added fifteen minims of water. A few drops were allowed to flow upon the gum of the upper jaw around the margins of the roots to be removed. After waiting about a minute, the needle was inserted along the buccal side of the first bicuspid, the point going to nearly on a level with the apex of the root, with scarcely any pain, forcing five minims out of the syringe, some of which entered an abscess sac. A similar quantity was injected into lingual gum, between second bicuspid and first molar, and the remaining five minims on the buccal aspect of gum, between first and second molars. After waiting about two minutes for absorption and the effects of the cocaine, I removed the roots of first and second bicuspids; the three roots of first molar; attempted the extraction of second molar, first with root forceps, then with ordinary molar forceps, and again with root forceps, bringing away the two buccal roots together, and afterwards the palatine root. All this was done leisurely; the patient rinsed the mouth with water on three occasions, and yet the whole operation was, according to the girl's evidence and, appearances, without pain. There being an immense abscess over the roots of the bicuspids, there was set free such a large quantity of blood and pus as to completely mask the roots behind. This was got rid of by washing the mouth as just mentioned, facilitating the remainder of the operation without marring the local anæsthesia, It is quite beyond possibility that I could have done so much with one administration of gas, or even gas and ether, and a second administration was, from the nature of the case, out of the question.

Cocaine hypodermically injected, as here set forth, is an immense boon to both patient and practitioner; to the latter especially, for ample time is given, which is not the case when nitrous oxide gas is administered, for the extraction of broken down and difficult teeth; at the same time, the patient, being conscious, can help in the work. The maximum anæsthetic effects are said to be observed between five and eight minutes after injection; the anæsthesia to last from ten to fifteen minutes, and then gradually disappear.

Until more is known of the constitutional effects of cocaine, it would be wiser not to indiscriminately inject several grains into one individual during the same day. It would appear that the alkaloid, when given to a certain extent, stimulates the sympathetic, increasing the heart's action and the blood pressure, with dilatation of the pupils. Among its toxic effects there have been noted sensations of coldness, paleness of the body, giddiness, uncertain gait, and a condition resembling alcoholic intoxication.—*Dental Record.*

